# Severe carotid artery stenosis evaluated by ultrasound is associated with post stroke vascular cognitive impairment

**DOI:** 10.1002/brb3.606

**Published:** 2016-11-22

**Authors:** Xuefeng Li, Xiangling Ma, Jing Lin, Xiangqin He, Feng Tian, Dongmei Kong

**Affiliations:** ^1^ Department of UltrasonographyJining No.1 People's HospitalJiningShandongChina; ^2^ Health Supervision InstituteZoucheng Municipal Health BureauZouchengShandongChina; ^3^ Department of NeurologyJining No.1 People's HospitalJiningShandongChina

**Keywords:** carotid artery stenosis, mini‐mental state examination score, stroke, ultrasound, vascular cognitive impairment

## Abstract

**Background:**

Acute ischemic stroke has been recognized as one key cause of vascular cognitive impairment (VCI). The purpose of this study was to evaluate the correlation between carotid artery stenosis and post VCI in acute ischemic stroke patients.

**Methods:**

In this study, B‐mode ultrasound was applied to measure the degree of carotid artery stenosis. After 1 year, the stroke patients’ cognitive function was assessed by the mini‐mental state examination (MMSE) score. The relationship between the VCI and degree of carotid artery stenosis was evaluated by multivariate regression analysis.

**Results:**

VCI was observed in 136 (37.2%) of the 365 participants. High degree of carotid artery stenosis was significantly correlated with VCI (*p* < .01), and this correlation remained unchanged even adjustment for age, gender, education level, stroke features, and vascular risk factors.

**Conclusions:**

These findings indicate that high‐grade stenosis of carotid artery is positively correlated with post stroke VCI in patients with acute ischemic stroke. The evaluation of 1 year post stroke cognitive function may be a potential tool for screening stroke patients at risk of VCI.

## 1 Introduction

Number of studies have reported that cerebral vascular disease is associated with cognitive function and shows a key role in the decline of memory (Feliziani et al., 2010; Lehrner et al., 2005; Xu, Liu, Meyer, Yin, & Zhang, 2007). It has been recognized that vascular cognitive impairment (VCI) is correlated with disability worldwide and is the most common manifestation of severe cerebrovascular disease (Hachinski, 2007; Kearney‐Schwartz et al., 2009; Sachdev et al., 2014). Notably, stroke may happen with risk factors of vascular disease and cause decline of cognitive function (Dempsey, Vemuganti, Varghese, & Hermann, 2010; Elias et al., 2004). VCI is one of the consequence of stroke affecting and has been found in 25%–75% stroke patients (Haring, 2002; Mlekusch et al., 2008; Zhou et al., 2009). These studies indicate that there are connections between cerebrovascular disease and post stroke VCI.

Accumulating evidence showed that patients with severe carotid artery stenosis are correlated with VCI in memory (Inzitari et al., 2000; Popovic et al., 2011; Sztriha, Nemeth, Sefcsik, & Vecsei, 2009). Patients with high‐grade carotid artery disease showed lower scores on cognitive function tests than normal controls (Sander et al., 2010; Silvestrini et al., 2011; Zhong et al., 2011, 2012). Haley et al. (2007) reported that carotid artery stenosis was associated with worse neuropsychological performance. Recent study showed that both symptomatic and asymptomatic carotid stenosis are associated with cognitive impairment (Wang, Mei, & Zhang, 2016). In contrast, previous epidemiological reports showed the correlations between intima‐media thickness of carotid and VCI in patients without stroke (Mathiesen et al., 2004; Pettigrew, Thomas, Howard, Veltkamp, & Toole, 2000). Interestingly, recent report by Yue et al. (2016b) demonstrated that intima‐media thickness of carotid was positively correlated with VCI in acute ischemic stroke patients and patients with cognitive impairment had a higher proportion of large artery stroke when compared to patients with good cognition. However, most articles reported outcomes within the first 6 months after stroke onset, and few studies investigated the correlations between the grade of carotid stenosis and 1 year post stroke VCI in patients with a major stroke.

Therefore, to better understand the correlation of post VCI and carotid artery stenosis, in this study, we explored the association of carotid artery stenosis with 1 year follow‐up post stroke VCI in stroke patients. The assessment of 1 year post stroke cognitive function may help to screen stroke patients at risk of VCI.

## 2 Materials and Methods

### 2.1 Study population

In this study, 382 consecutive Chinese participants with first ever stroke were enrolled from January 2013 to December 2014 in Jining No.1 People's Hospital. The first ever stroke were defined based on the criteria of WHO (Aho et al., 1980). Imaging of brain was available for all patients. The inclusion criteria was: (1) patients age older than 18 years; (2) the diagnosed stroke was confirmed by magnetic resonance imaging (MRI) or computed tomography (CT); (3) less than 1 week from the first ever stroke; (4) the informed consent was provided by participant. The criteria of exclusion was: (1) other diseases on brain, including tumors, vascular malformations, abscesses, or multiple sclerosis; (2) transient ischemic attack; (3) medical treatment during the study; (4) patients without carotid artery stenosis; (4) patients had previous cognitive impairment. This is a double blind retrospective study. This research was approved by the Ethics Committee of Jining No.1 People's Hospital and all study participants provided the informed written consent.

### 2.2 Data collection

Information regarding demographic data (alcohol use, age, education, sex, history of disease), vascular risk factors and past medical history were recorded via questionnaires at time of stroke onset. Intake 100 ml of liquor per day three times 1 week for at least 1 year was defined as alcohol use. The physical activity both during leisure time and at work was evaluated. Previous history of diseases, including stroke, myocardial infarction, diabetes, hypertension, atrial fibrillation, coronary artery disease, and hypercholesterolemia were recorded via self‐reporting. The past medical history of using antihypertensive, glucose lowering and cholesterol lowering medications <2 weeks prior to the baseline interview was also recorded through self‐reporting.

A mercury sphygmomanometer was applied to measure blood pressure. Two readings (5 min interval) of blood pressure were obtained. To research whether the serum lipid profile and blood glucose levels are associated with participants’ cognitive status, phlebotomists draw blood from the patients after overnight fasting. Blood samples were centrifuged and the serum was obtained. Serum levels of fasting blood glucose (FBG), low‐density lipoprotein (LDL), total cholesterol (TC), and high‐density lipoprotein cholesterol (HDL) were determined by an autoanalyzer (Olympus, AU400, Japan).

### 2.3 Ultrasound examination

All participants were examined by carotid ultrasound during hospitalization. One experienced operator did all the measurements using B‐mode ultrasound according to the Atherosclerosis Risk in Communities protocol (Li et al., 1994; Riley et al., 1991). The ultrasound measurement included scanning the left and right carotid arteries, bifurcations, and internal carotid arteries (the first 2 cm). The density of plaques was also examined. The grade of carotid artery stenosis was classified in two groups according to previous report (Faught et al., 1994): low‐grade stenosis (<70%) and high‐grade stenosis (≥70%), according to peak systolic velocity of the internal carotid/common carotid artery.

### 2.4 Follow‐up and neuropsychological evaluation

The cognitive function of the participants was measured using the Mini Mental State Examination (MMSE) by one investigator blinded to clinical characteristics of participants. The MMSE measurement includes orientation to place and time, calculation and attention, memory, and language. Higher score indicates better cognitive function. Cognitive dysfunction is considered as MMSE scores <24 (Yue et al., 2016a).

### 2.5 Statistical analysis

All statistical analysis was performed by Prism (GraphPad Software, Inc.). Simple descriptive statistics were calculated using means and standard deviations for continuous data and percentages for categorical data. The data dispersion was checked before analysis. Differences between groups were determined by using unpaired *t* test (two tailed) and Chi square test or Fisher's exact test. Logistic regression analysis was performed for categorical‐dependent variables. *p* values of <.05 were regarded as statistically significant.

## 3 Results

Of the 382 participants who were initially registered in this study, 17 patients were excluded as not meeting requirements during the follow‐up period. The remaining participants (*n* = 365) were evaluated at 1 year later after stroke; neuropsychological assessment data and ultrasound images were obtained. Clinical characteristics of participants categorized by cognitive function status were summarized in Table 1. Among the 365 participants, 136 (37.3%) were diagnosed with VCI, which was defined as an MMSE score <24, and 229 (62.7%) participants were determined with good cognition. Older age (*p* < .01, *t* = 7.8, *R* ^2^ = .14), lower education level (*p* < .01), and history of atrial fibrillation (*p* < .01) were correlated with decline of cognitive function. Moreover, participants with VCI exhibited a higher likelihood of high grade of carotid stenosis compared to good cognition (25.7% vs. 14.4%, *p* < .01). Similarly, compared with good cognitive, participants with VCI showed a higher proportion of large artery stroke (*p* < .01), while no significant difference was found in cardioembolism stroke. The serum levels of TC, FBG, HDL, and LDL were also determined, however, no difference was observed between good cognitive and VCI groups. Further, there were no significant differences among other general characteristics of participants including gender, marital status, weight, blood pressure, alcohol use, history of hypertension, diabetes, hypercholesterolemia, and coronary artery disease.

**Table 1:** The general characteristics of participants (*n* = 365) stratified by cognitive status

	Cognitively not impaired (MMSE ≥ 24) *n* = 229	Cognitively impaired (MMSE<24) *n* = 136	*p* value	Other statistical values
Age (mean ± *SD*)	61.3 ± 11.8	71.9 ± 13.6	<.01	*t* = 7.8, *R* ^2^ = .14
Male, *n* (%)	114 (49.8%)	72 (52.9%)	.67	
Married, *n* (%)	218 (95.2%)	131 (96.3%)	.70	
BMI (kg/m^2^)	23.6 ± 4.8	24.1 ± 5.7	.37	
SBP, mm Hg	143.2 ± 21.5	146.7 ± 24.3	.15	
DBP, mm Hg	87.1 ± 13.4	85.7 ± 12.3	.32	
Lipid profile and glucose levels				
TC, mmol/L	4.8 ± 1.4	4.9 ± 1.7	.54	
FBG, mmol/L	6.4 ± 2.2	6.7 ± 2.5	.23	
HDL, mmol/L	1.3 ± 1.1	1.5 ± 1.2	.10	
LDL, mmol/L	2.7 ± 1.2	2.9 ± 1.3	.14	
Alcohol use (Yes)	87 (38.0%)	55 (40.4%)	.66	
Tobacco use (Yes)	96 (41.9%)	50 (36.8%)	.47	
Education level (years of schooling)				
0–5, *n* (%)	52 (22.7%)	81 (59.6%)	<.01	OR = 0.19, CI = 0.10–0.36
≥6, *n* (%)	177 (77.3%)	55 (40.4%)		
History of diseases, *n* (%)				
Hypertension	156 (68.1%)	96 (70.6%)	.76	
Diabetes	53 (23.1%)	36 (26.5%)	.62	
Hypercholesterolemia	71 (31.0%)	35 (25.7%)	.34	
Atrial fibrillation	32 (14.0%)	39 (28.7%)	<.01	
Coronary artery disease	14 (6.1%)	10 (7.4%)	.77	
Stroke subtypes, *n* (%)				
Large artery stroke	123 (53.7%)	97 (71.3%)	<.01	OR = 0.45, CI = 0.25–0.82
Supratentorial cardioembolic	16 (7.0%)	13 (9.6%)	.42	
Supratentorial atherothrombotic	15 (6.6%)	10 (7.4%)	.77	
Affected cerebral artery *n* (%)				
Middle cerebral artery	96 (41.9%)	63 (46.3%)	.47	
Anterior cerebral artery	61 (26.6%)	41 (30.1%)	.53	
Posterior cerebral artery	72 (31.4%)	32 (23.5%)	.20	
Degree of carotid stenosis				
<70%	196 (85.6%)	35 (25.7%)	<.01	OR = 18.0, CI = 8.7–37.1
≥70%	33 (14.4%)	101 (74.3%)		

*SD*, standard deviation; MMSE, mini‐mental state examination; SBP, systolic blood pressure; DBP, diastolic blood pressure; TC, total cholesterol; FBG, fasting blood glucose; HDL, high‐density lipoprotein cholesterol; LDL, low‐density lipoprotein cholesterol; BMI, body mass index; OR, odds ratio; CI, 95% confidence interval.

To determine the relationships between the grade of carotid artery stenosis and clinical characteristics, the participants with low‐grade carotid stenosis (<70%, *n* = 297) and high‐grade carotid stenosis (≥70%, *n* = 68) were compared. There were no significant differences among alcohol use, tobacco use, gender, education level, marital status, weight, atrial fibrillation, diabetes, hypertension, coronary artery disease, hypercholesterolemia, supratentorial cardioembolic or atherothrombotic stroke, and affected cerebral artery in participants with low or high grade of carotid artery stenosis, while the age (*p* < .01, *t* = 6.7, *R* ^2^ = .12) and large artery stroke (*p* < .01) were observed to be significantly different in those patients (Table 2).

**Table 2:** Clinical characteristics stratified by degree of carotid stenosis

	Low‐grade carotid stenosis (<70%) *n* = 297	High‐grade carotid stenosis (≥70%) *n* = 68	*p* value	Other statistical values
Age (mean ± *SD*)	60.5 ± 10.4	73.1 ± 14.2	<.01	*t* = 6.7, *R* ^2^ = .12
Male, *n* (%)	153 (51.5%)	33 (48.5%)	.78	
Married, *n* (%)	286 (96.3%)	63 (92.6%)	.33	
BMI (kg/m^2^)	23.3 ± 6.2	24.5 ± 5.1	.14	
SBP, mm Hg	142.4 ± 29.6	148.6 ± 25.7	.11	
DBP, mm Hg	84.2 ± 14.1	86.8 ± 13.2	.16	
Lipid profile and glucose levels				
TC, mmol/L	4.5 ± 1.2	4.7 ± 1.8	.26	
FBG, mmol/L	6.5 ± 2.4	6.6 ± 2.8	.76	
HDL, mmol/L	1.2 ± 1.0	1.4 ± 1.3	.16	
LDL, mmol/L	2.5 ± 1.3	2.7 ± 1.2	.25	
Alcohol use (Yes)	114 (38.4%)	28 (41.2%)	.78	
Tobacco use (Yes)	122 (41.1%)	24 (35.3%)	.48	
Education level (years of schooling)				
0–5, *n* (%)	111 (37.4%)	22 (32.4%)	.55	
≥6, *n* (%)	186 (62.6%)	46 (67.6%)		
History of diseases, *n* (%)				
Hypertension	206 (69.4%)	46 (67.6%)	.88	
Diabetes	72 (24.2%)	17 (25.0%)	1.0	
Hypercholesterolemia	85 (28.6%)	21 (30.9%)	.87	
Atrial fibrillation	56 (18.9%)	15 (22.1%)	.59	
Coronary artery disease	20 (6.7%)	4 (5.9%)	1.0	
Stroke subtypes, *n* (%)				
Large artery stroke	165 (55.6%)	55 (80.9%)	<.01	OR = 0.30, CI = 0.16–0.56
Supratentorial cardioembolic	23 (7.7%)	6 (8.8%)	1.0	
Supratentorial atherothrombotic	18 (6.1%)	7 (10.3%)	.44	
Affected cerebral artery *n* (%)				
Middle cerebral artery	127 (42.8%)	32 (47.1%)	.57	
Anterior cerebral artery	81 (27.3%)	21 (30.9%)	.75	
Posterior cerebral artery	89 (30.0%)	15 (22.1%)	.33	

*SD*, standard deviation; MMSE, mini‐mental state examination; SBP, systolic blood pressure; DBP, diastolic blood pressure; TC, total cholesterol; FBG, fasting blood glucose; HDL, high‐density lipoprotein cholesterol; LDL, low‐density lipoprotein cholesterol; BMI, body mass index; OR, odds ratio; CI, 95% confidence interval.

We further analyzed the associations between cognitive function and different degree of carotid stenosis using multivariate regression analysis. As seen in Table 3, after adjusting for potential confounders, including age, sex, marriage, alcohol use, education, tobacco use, diabetes, atrial fibrillation, hypertension, large artery stroke, hypercholesterolemia, and cardioembolism measurements at 1 year post stroke, the participants with high‐grade carotid stenosis showed a significant higher frequency of having VCI compared to patients with low‐grade carotid artery stenosis (*p* < .01).

**Table 3:** Odds ratio of cognitive impairment at 1 year post‐stroke in the multivariate logistic regression analysis based on degree of carotid artery stenosis

	Odds ratio (95% CI) by degree of carotid artery stenosis		*p* value for trend
	High‐grade carotid stenosis (≥70%) *n* = 68	Low‐grade carotid stenosis (<70%) *n* = 297	
Case number	68	297	
Model 1	2.61 (1.42–2.95)	1.16 (0.78–1.52)	<.001
Model 2	2.13 (1.57–3.26)	1.04 (0.72–1.48)	<.001

CI, confidence interval.

Model 1: Adjusted for age (years), sex, marriage. Model 2: Adjusted for as model 1 plus education level, alcohol use, tobacco use, hypertension, diabetes, hypercholesterolemia, atrial fibrillation, large artery stroke and cardioembolism.

## 4 Discussion

This study investigated the relationship between the grade of carotid stenosis and cognitive disfunction in ischemic stroke patients. Our results showed a difference in MMSE between patients with a stenosis grade of <70% and patients with a stenosis grade of >70%, indicating that acute ischemic stroke patients who had higher grade carotid artery stenosis were at high likelihood of post stroke cognitive disfunction. The association between VCI and severe carotid stenosis persisted even after adjusting for potential confounders.

It has been well known that carotid artery stenosis disease was correlated with decline of cognitive function in patients without stroke (Cerhan et al., 1998). In addition, another study showed that increased carotid artery intima‐media thickness was linked with cognitive disfunction in patients with atherosclerotic plaques (Auperin et al., 1996). In this study, we observed that older age, lower education level, and history of atrial fibrillation were correlated with decline of cognitive function. Similarly, compared with good cognitive, participants with VCI showed a higher proportion of large artery stroke. These results are consistent with previous report that high‐grade carotid stenosis has been known as a predictor of VCI in a prospective study (Johnston et al., 2004). Talelli et al. (2004) also reported that the cognitive impairment of 1 year after stroke was independently correlated with higher common carotid artery intima‐media thickness. Interestingly, one recent study showed that cognitive disfunction was correlated with severe right carotid artery stenosis (Yue et al., 2016a). In this study, we also found that severe carotid stenosis was positive associated with VCI. We showed a difference in MMSE between patients with a stenosis grade of <70% and patients with a stenosis grade of >70%. These results suggest that severe carotid stenosis have important effect on VCI. Additionally, it has been demonstrated that cerebral hypoperfusion caused by severe carotid stenosis was correlated with decline of cognitive function (Consoli, Pasi, & Pantoni, 2012). Thus, these findings highlight the potential value of severe carotid stenosis as a potential predictor of VCI in patients with acute stroke.

In this study, to evaluate the relationships between the grade of carotid artery stenosis and clinical characteristics, the participants with low‐grade carotid stenosis (<70%) and high‐grade carotid stenosis (≥70%) were compared. The age and large artery stroke were observed to be significantly different in those patients. Our findings are in accordance with the results of previous works (Desmond et al., 2000; Patel, Coshall, Rudd, & Wolfe, 2002; Pohjasvaara et al., 1998; Rockwood, 2002). In contrast, Auperin et al. (1996) demonstrated that carotid plaques were correlated with VCI only in male. In addition, diabetes mellitus, cardiac disease, arterial hypertension, and smoking have also been correlated with cognitive decline (Desmond et al., 2000; Elwood, Pickering, Bayer, & Gallacher, 2002; Knopman et al., 2001; Rao, 2002), but in this study, no significant correlation was observed among alcohol use, tobacco use, gender, education level, marital status, atrial fibrillation, diabetes, hypertension, coronary artery disease, hypercholesterolemia, and cardioembolism stroke in patients with low or high grade of carotid artery stenosis. However, it has been acknowledged that the comparison of clinical cognitive function data is often limited by different diagnostic tool, different criteria of inclusion and exclusion, and variation interval between the index of stroke and the assessment of cognitive function (Auperin et al., 1996). We further analyzed the associations between cognitive function and different degree of carotid stenosis using multivariate regression analysis. We found that after adjusting for potential confounders, the participants with high‐grade carotid stenosis showed a significant higher frequency of having VCI compared to patients with low‐grade carotid artery stenosis. The possible reason for the correlations between severe carotid stenosis and VCI was also considered in this study. The correlation between carotid disease and VCI was first reported by Fisher that the increasing cerebral perfusion caused by reopening internal carotid arteries may have an positive effect on cognitive function(Fisher, 1951). Several other reports have also indicated that cognition was improved in some patients with endarterectomy (Greiffenstein, Brinkman, Jacobs, & Braun, 1988; Hemmingsen, Mejsholm, Boysen, & Engell, 1982; Jacobs, Ganji, Shirley, Morrell, & Brinkman, 1983), whereas some reports have not shown such effect (Casey, Ferguson, Kimura, & Hachinski, 1989; Pettigrew et al., 2000; Sirkka, Salenius, Portin, & Nummenmaa, 1992). Therefore, it could be better to evaluate VCI in future studies of therapy for carotid artery stenosis disease.

The limitations of this study should also be considered. First, cognitive dysfunction in this study was assessed by the MMSE, which could evaluate amnestic cognitive patients but not the non‐amnestic ones. To better characterize the pattern of “cognitive dysfunction” from patients, more assessment methods including Montreal Cognitive Assessment (MoCA), Rey Auditory Verbal Learning Test (RAVLT), Verbal fluency (letters, animals), line cancelation, short‐15 items from the Boston Naming Test are needed in the future study. Second, the number of participants was relatively low. Therefore, the analysis should be repeated in larger cohort studies. Third, only Chinese participants were included in this study, thus, our findings may not be fully applied to the common population.

In conclusion, we demonstrated a difference in MMSE between patients with a stenosis grade of <70% and patients with a stenosis grade of >70%, suggesting that acute stroke patients who had severe carotid stenosis were at high risk of post stroke VCI. This relationship persisted even the potential confounders were adjusted. The evaluation of cognitive function may be a potential adjunct tool for screening post stroke patients at high risk of VCI.

## Conflict of Interests

The authors declare that they have no conflict of interest.
